# Drinking-Water Arsenic Exposure Modulates Gene Expression in Human Lymphocytes from a U.S. Population

**DOI:** 10.1289/ehp.10861

**Published:** 2008-01-23

**Authors:** Angeline S. Andrew, David A. Jewell, Rebecca A. Mason, Michael L. Whitfield, Jason H. Moore, Margaret R. Karagas

**Affiliations:** 1 Department of Community and Family Medicine, Section of Biostatistics and Epidemiology and; 2 Department of Genetics, Dartmouth Medical School, Lebanon, New Hampshire, USA

**Keywords:** arsenic, drinking water, immune response, lymphocytes, microarray, U.S. population

## Abstract

**Background:**

Arsenic exposure impairs development and can lead to cancer, cardiovascular disease, and diabetes. The mechanism underlying these effects remains unknown. Primarily because of geologic sources of contamination, drinking-water arsenic levels are above the current recommended maximum contaminant level of 10 μg/L in the northeastern, western, and north central regions of the United States.

**Objectives:**

We investigated the effects of arsenic exposure, defined by internal biomarkers at levels relevant to the United States and similarly exposed populations, on gene expression.

**Methods:**

We conducted separate Affymetrix microarray-based genomewide analyses of expression patterns. Peripheral blood lymphocyte samples from 21 controls interviewed (1999–2002) as part of a case–control study in New Hampshire were selected based on high- versus low-level arsenic exposure levels.

**Results:**

The biologic functions of the transcripts that showed statistically significant abundance differences between high- and low-arsenic exposure groups included an overrepresentation of genes involved in defense response, immune function, cell growth, apoptosis, regulation of cell cycle, T-cell receptor signaling pathway, and diabetes. Notably, the high-arsenic exposure group exhibited higher levels of several killer cell immunoglobulin-like receptors that inhibit natural killer cell activity.

**Conclusions:**

These findings define biologic changes that occur with chronic arsenic exposure in humans and provide leads and potential targets for understanding and monitoring the pathogenesis of arsenic-induced diseases.

Arsenic exposure impairs development and can lead to cancer, cardiovascular disease, and diabetes [International Agency for Research on Cancer (IARC) 2004]. The mechanism underlying these effects remains unknown. Primarily because of geologic sources of contamination, drinking-water arsenic levels are above the current recommended maximum contaminant level of 10 μg/L in several areas of the United States, including levels exceeding 20 μg/L in 12% of water supplies from surface water sources in the north central region, and ground-water sources in the West [Agency for Toxic Substances and Disease Registry (ATSDR) 2007; Mead 2005]. Approximately 40% of households in New Hampshire are served by unregulated private drinking-water wells, and 10% of these wells contain arsenic at levels > 10 μg/L (Karagas et al. 2002; Mead 2005).

Although many laboratory-based animal or cell-culture studies have investigated the effects of arsenic exposure on gene expression using microarray analysis, many of these were conducted on arsenic exposures outside the normal range of U.S. contamination, and only a few have studied arsenic-exposed humans (Andrew et al. 2007; Argos et al. 2006; Lu et al. 2001; Wu MM et al. 2003). Progress in arsenic research has been hampered by the wide variations in its dose–response effects across different species and cell lines (IARC 2004). Humans are believed to be more sensitive to the toxic effects of arsenic than are model organisms; therefore it is important to understand the exposure implications on a genomewide scale (Mead 2005). The first two human studies of arsenic exposure employed smaller arrays representing < 1,000 genes to examine arsenic-exposed tissues. Lu et al. (2001) used liver biopsies from China (*n* = 6), whereas Wu MM et al. (2003) used peripheral blood lymphocytes from 24 subjects to compare the effects of low (0–46.4 μg/dL) versus high (46.4–465 μg/dL) blood arsenic levels [normal blood arsenic levels are around 70 μg/dL (ATSDR 2007)]. Recently, Argos et al. (2006) used peripheral blood lymphocytes from a population of highly exposed individuals from Bangladesh with skin lesions and the Affymetrix GeneChip microarray platform to investigate differentially expressed genes associated with arsenic exposure. Mean well-water levels of arsenic in the Bangladesh study were 342.7 μg/L for the high-exposure group (*n* = 11) and 39.6 μg/L for the low-exposure group (*n* = 5), which is well above the current maximum contaminant level of 10 μg/L.

Thus, the objective of the present study was to investigate the effects of arsenic exposure at levels relevant to North American populations. We chose high- versus low-arsenic-exposed groups from a population in New Hampshire, where the rural landscape results in 40% of the population consuming drinking water from unregulated private wells (Karagas et al. 2002, 2004).

We conducted a microarray-based genome-wide analysis of expression patterns associated with internal biomarkers of arsenic exposure in peripheral blood lymphocytes from the high-versus low-exposure groups for this population. Our analysis characterizes the major biologic functional classes of genes differentially expressed in arsenic-exposed individuals. Understanding the affected biologic pathways will guide investigations of carcinogenic and pathogenic mechanisms and assist in the development of remediation techniques and chemo-preventive agents for exposed individuals.

## Materials and Methods

### Subject selection

This project used samples from an epidemiologic case–control study in New Hampshire described previously (e.g., Karagas et al. 1998, 2004). One of our major goals of this study was to investigate the bladder and nonmelanoma skin cancer risks associated with arsenic exposure in New Hampshire. Controls younger than 65 years were selected using population lists obtained from the New Hampshire Department of Transportation. Controls 65 or more years of age were chosen from data files provided by the Centers for Medicare and Medicaid Services of New Hampshire. Subjects were sent an introductory recruitment letter followed by a phone call to arrange an in-person interview. For this project, we selected subjects from a group of 606 controls who were interviewed between 1999 and 2002. The overall study participation rate was approximately 70% of the controls confirmed to be eligible for the study. Of those from whom we requested samples, the participation rate was approximately 72% for the blood draw, 90% for toenail clippings, 95% for urine collection, and more than 95% for water. Data on subjects’ exposure history were available through a personal interview covering demographic information, history of tobacco use, and other lifestyle factors. Informed consent was obtained from each participant, and all procedures and study materials were approved by the Committee for the Protection of Human Subjects at Dartmouth College.

Selection of the 21 control subjects (i.e., who did not have cancer) who were used for this present project was based on internal biomarkers (toenail or urine) and household drinking-water arsenic exposure levels from individuals on whom cryopreserved lymphocytes were available at the time the project began. The selected subjects had agreed to provide a venous blood sample that was drawn into cell preparation tubes (CPTs) containing citrate and a lymphocyte isolation gradient (Becton, Dickinson and Co., Franklin Lakes, NJ). Blood tubes were maintained at 4°C and sent to the study laboratory for processing and analysis. No later than 24 hr after the blood draw, lymphocytes collected in CPTs containing sodium citrate were isolated according to the manufacturer’s instructions using standard buoyant density centrifugation methods. After centrifugation, first plasma was removed, aliquoted, and frozen at −80°C, then the mononuclear cells were removed by pipette and cryopreserved (−120°C) using freezing media at a controlled rate of 1°C per minute. The viability of the cells after thawing was assessed to be 98% using trypan blue, as reported previously for this method (Wei et al. 1994).

A water sample from the current household drawn into commercially washed (mineral-free) high-density polyethylene bottles that met U.S. Environmental Protection Agency standards for water collection (I-Chem vials; Fisher Scientific, Pittsburgh, PA) was analyzed for arsenic concentration using an Agilent 7500c Octopole inductively coupled plasma mass spectrometer (Agilent Technologies, Palo Alto, CA) in the Dartmouth Trace Element Analysis Core Facility. Toenail clipping samples collected at the time of interview were analyzed for arsenic and other trace elements by instrumental neutron activation analysis (INAA) at the University of Missouri Research Reactor, using a standard comparison approach as described previously (Cheng et al. 1995). The detection limit for arsenic measured by INAA is approximately 0.001 μg/g. First morning void urine samples were obtained in 100-mL polypropylene bottles and kept on ice. Within 6 hr, cooled samples were taken to the laboratory and kept frozen at −80°C until the analysis of total arsenic and arsenic species was performed as described previously (Meza et al. 2005). The detection limits were 0.42–1.08 μg/L for arsenic compounds.

Individuals from whom we had collected cryopreserved lymphocyte, drinking-water, and either toenail or urine samples were candidates for the microarray project. From this subset, we first selected individuals for the high-arsenic group based on arsenic exposure criteria defined by drinking-water arsenic concentration combined with urinary or toenail arsenic levels as internal biomarkers of exposure, as detailed below. We then selected a set of low arsenic exposure subjects matched for age, sex, and smoking status. Within the subset selected for the microarray project, the drinking-water arsenic levels of the high-exposure group (*n* = 11) averaged 32 μg/L (range, 10.4–74.7 μg/L), whereas the levels for the low-exposure group (*n* = 10) averaged 0.7 μg/L (range, 0.007–5.3 μg/L). Individuals with inorganic urinary arsenic levels (overnight > 5 μg/L, spot > 1 μg/L) or > 0.11 μg/g toenail arsenic were considered to have high arsenic exposure.

### Gene expression analysis

**Figure 1:**
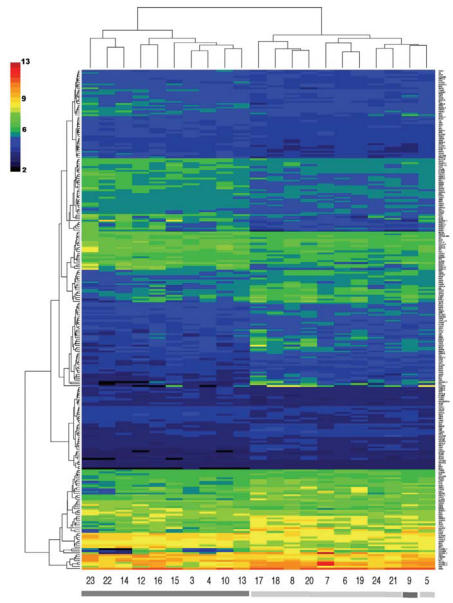
Heat map of genes with statistically significant expression differences by arsenic exposure level. The rows represent the genes selected by SAM analysis. The heat-map colors depict the gene expression level from low (black) to high (red). Each column represents an individual (numbered at the bottom) with either high (dark gray bar at the bottom) or low (light gray) arsenic exposure level. Genes and individuals were hierarchically clustered by expression level.

RNA was harvested from peripheral blood lymphocytes using Trizol reagent (Gibco/BRL Life Technologies, Gaithersburg, MD) followed by DNase digestion using DNAfree (Ambion Inc., Austin, TX) according to the manufacturer’s instructions and quantitated by spectrophotometric absorbance at 260 nm. RNA quality was evaluated using A260/A280 ratio (> 1.8) and the RNA 6000 Nano Chips in the Agilent 2100 Bioanalyzer (Agilent Technologies). The expression profiles were generated using the Affymetrix GeneChip Technology Human Genome U133 Plus 2.0 oligonucleotide arrays (Affymetrix, Santa Clara, CA), which simultaneously tested more than 47,000 transcripts for each subject on the integrated GeneChip Instrument System in the Dartmouth Microarray Core Facility. Our experiment was performed in compliance with the Minimum Information About a Micro-array Experiment (MIAME) checklist for standardization guidelines for microarray experiments. Array data from this experiment will be available on the National Institutes of Health GEO database or by contacting the author. Affymetrix chip CEL files were imported into the statistical programming language R and analyzed using the Bioconductor package “affy” (Gentlemen et al. 2005). Data were normalized using robust multichip analysis (RMA) implemented with Bioconductor software (Irizarry et al. 2003) followed by empirical Bayes adjustment procedures implemented on the R platform, as described previously (Johnson et al. 2007). Analysis using the Statistical Analysis of Microarrays (SAM) package (Stanford University, Stanford, CA; http://www-stat.stanford.edu/~tibs/SAM) was performed with a two-class comparison between the high- versus low-arsenic exposure groups, defined as described above by drinking-water arsenic concentration combined with urinary and toenail arsenic levels. We used 1,000 permutations and selected significant genes with a false discovery rate (FDR) of < 5% from a delta value of 0.7 to identify statistically significant differences in gene expression, accounting for multiple comparisons (Tusher et al. 2001). We constructed a heat map (Figure 1) for these 259 genes, and their successful agglomerative hierarchical clustering into high- and low-arsenic-exposed groups was assessed via bootstrap evaluation with confidence levels of > 98% [for a complete list, see Supplemental Material (http://www.ehponline.org/members/2008/10861/suppl.pdf)] (Kamimura et al. 2003).

We performed an additional analysis restricted to a subset of 10 individuals with urinary arsenic exposure data as an internal measure of very recent arsenic exposure from the originally selected set of 21 subjects (Biggs et al. 1997). This additional analysis excluded subjects on whom we did not have urinary arsenic data, because toenail arsenic reflects chronic exposure (previous 12–18 months) (Slotnick and Nriagu 2006). Microarray data on subjects with urinary arsenic levels were normalized using RMA implemented with Bioconductor software on the R platform (Irizarry et al. 2003). Subsequent SAM analysis using a delta of 0.2 yielded 38 modified genes at or below 5% FDR for the high- versus low-arsenic-exposure groups [for a list, see Supplemental Material (http://www.ehponline.org/members/2008/10861/suppl.pdf)].

We selected transcripts for validation by real-time polymerase chain reaction (PCR) using independent primer sets based on the microarray results. TaqMan primer-probe sets for each selected transcript were obtained from Applied Biosystems Inc. (Foster City, CA): perforin 1 (*PRF1*), interleukin 2 receptor, beta (*IL2RB*), killer cell immunoglobulin-like receptor, three domains, long cytoplasmic tail, 1 (*KIR3DL1*), and major histocompatibility complex (*MHC*), class II, DR beta 1 (*HLA-DRB1*). Real-time reverse transcription (RT)-PCR was performed using the Applied Biosystems Inc. PRISM sequence detection system and software. Briefly, total RNA (1.0 μg) was reverse transcribed using 100 U Moloney murine leukemia virus reverse transcriptase in a mixture with oligo-dT and dNTPs according to the instructions provided with the Omniscript kit (QIAGEN Inc., Valencia, CA). Samples were reverse transcribed in a PTC-100 thermocycler (MJ Research Inc., Watertown, MA) for 60 min at 44°C, and the reaction was terminated by heating to 95°C for 10 min. Expression of specific genes was assessed by real-time PCR using 10 ng total RNA, 400 nM primers, 200 nM probe, and TaqMan Universal PCR Master Mix (Applied Biosystems Inc.). Relative quantitation was performed using a standard curve consisting of serial dilutions of pooled sample cDNA from the same source as the test RNA with each plate. Relative expression levels of each gene were normalized to the cDNA concentrations and plotted against the creatinine-normalized urinary arsenic level.

### Biological function analysis

We further characterized the functional effects of the arsenic-modified genes by implementing the Database for Annotation, Visualization, and Integrated Discovery (DAVID) Gene Ontology (GO) search engine (http://www.geneontology.org/GO.tools.microarray.shtml; Dennis et al. 2003). This bioinformatic tool identifies functional processes that are overrepresented by the modified genes. Genes with statistically significant expression differences were also mapped to Kyoto Encyclopedia of Genes and Genomes (KEGG; http://www.genome.jp/kegg/pathway.html) biopathways, including the natural killer cell–mediated cytotoxicity pathway, to investigate their systemic roles.

**Figure 2:**
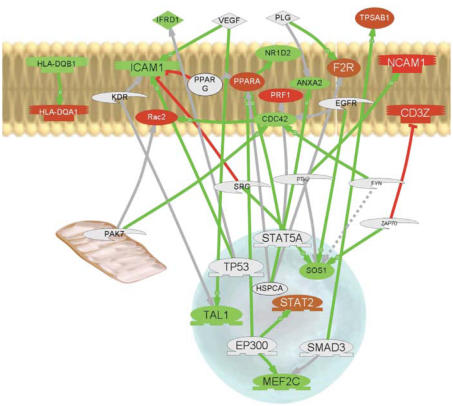
Pathway Studio diagram of common regulators of differentially expressed genes. Genes with statistically significant expression differences were queried against the Pathway Studio ResNet 5.0 database to identify common regulators. Biological relationships are represented by red arrows for positive effects and green arrows for negative effects. Genes are represented by colored shapes: those with increased expression with high arsenic exposure are shown in red; those with decreased expression in response to high arsenic exposure are shown in green. Other genes that are directly involved in the pathway, but were not significantly modified at the gene expression level by arsenic exposure status, are shown in gray.

Biologic roles of the arsenic-modified genes were then queried against the Pathway Studio ResNet 5.0 database (Ariadne Genomics, Rockville, MD). This database catalogs relationships between biologic entities based on the published literature and was used to identify the direct interactions between the arsenic-modified genes. These relations are depicted by colored lines and arrows in Figure 2.

We also implemented the Exploratory Visual Analysis (EVA) software package to facilitate graphical interpretation of our microarray data in the context of biological information, as described previously (Reif et al. 2005; Reif and Moore 2006). We prefiltered the normalized data from all 21 individuals using the ReliefF algorithm (Kononenko et al. 1997), and the top-ranked 12,484 genes were queried against KEGG biopathways. This strategy allowed us to explore broader trends in the data that may have been missed on the more restrictive SAM list.

## Results

To evaluate the possibility of gene expression modification by arsenic, we selected subjects according to individual exposure status from those who provided a blood sample. The high-and low-arsenic-exposure groups are comparable for sex (high arsenic: 82% male, 18% female; low arsenic: 80% male, 20% female) and age (high arsenic: mean, 66 years; low arsenic: mean, 67 years). The high-arsenic-exposure group had a slightly higher proportion of smokers (high arsenic: no, 73%, yes, 27%; low arsenic: no, 90%, yes, 10%). This selected subset is similar to the population of total controls for age, sex, and smoking status (Andrew et al. 2006).

We identified 259 genes with statistically significant expression differences (high vs. low arsenic exposure status in 21 individuals) by the SAM test [Figure 1; Supplemental Material, Table 1 (http://www.ehponline.org/members/2008/10861/suppl.pdf)]. The expression of the statistically significant genes reliably reproduced the dose–response clustering of subjects by arsenic exposure level (Figure 1, columns). With the exception of subject 9, all of the individuals exposed to high arsenic levels clustered separately (dark gray bars) from those exposed to low levels (light gray bars). Although there is some variability, as expected in a study of humans, the clustering of the rows (genes) on the heat map reveals distinct patterns of transcript abundance between lymphocyte samples that clearly separate the high- from the low-arsenic-exposed subjects (between subjects 13 and 17). We also performed a sensitivity analysis by running the algorithm with and without women or individuals who had a history of smoking. The genes with modified transcript abundance show similar patterns regardless of smoking status or sex.

Functional categories of these SAM-selected significant transcripts detected by DAVID functional annotation clustering indicated enrichment (*p* < 0.05) for genes involved in defense response (*p* < 0.001), immune response (*p* = 0.003), cell growth (*p* = 0.001), signal transduction (*p* = 0.01), apoptosis (*p* = 0.01), regulation of cell cycle (*p* = 0.02), JAK-STAT cascade (*p* = 0.004), T-cell receptor signaling pathway (*p* = 0.01), GTPase activity (*p* = 0.008), and type 1 diabetes mellitus (*p* = 0.002). The selected transcripts involved in diabetes included *PRF1*, *HLA-DQA1*, *HLA-DQB1*, *SOCS3*, *HLA-DPA1*, and *GZM2*. We also saw significant differences in transcripts involved in the nervous system (e.g., *MATK*, *NCAM1*, *GPR56*, *MEF2C*, *ZFHX1B*) and other aspects of development (e.g., *AES*, *TAGLN*, *ARIH2*, *WNT10A*, *MRAS*, *ITGA6*, *ANXA2*, *VAMP5*) [Supplemental Material, Table 1 (http://www.ehponline.org/members/2008/10861/suppl.pdf]. In the ubiquitin cycle, we saw differences between high- and low-arsenic groups for F-box protein transcripts *FBXO32*, *FBX03*, *TRIAD3*, and ariadne homolog 2 (*ARIH2*).

The pathways with the most statistically significant level of enrichment and largest number of modified transcripts associated with arsenic exposure were those involved in defense and immune response (listed in Table 1). These included induction of many probe sets that detected various isoforms of the killer cell immunoglobulin-like receptor that inhibits the activity of the MHC class I receptor. A number of the SAM-selected genes that showed increased or decreased transcript abundance in lymphocytes from the high- versus low-exposure groups are part of the natural killer cell cytotoxic KEGG pathway [Supplemental Material, Figure 1 (http://www.ehponline.org/members/2008/10861/suppl.pdf)]. Notably, expression of the killer cell immunoglobulin-like inhibitory receptors (KIR) is increased. Arsenic exposure was also associated with modified levels of granzyme b (*GZMB*) and *PRF1*, enzymes that control natural killer cell–mediated apoptosis. Our data suggest decreases in defense response genes, including the heat-shock protein *HSPA9B*, *CD69*, and mucosa associated lymphoid tissue lymphoma translocation gene 1 (*MALT1*) associated with arsenic exposure. Inflammatory response pathway members selected in our analysis included *IL2RB*, carbohydrate (*N*-acetylglucosamine-6-*O*) sulfotransferase 2 (*CHST2*), nuclear factor of activated T-cells (*NFATC3*), arachidonate 5-lipoxygenase (*ALOX3*), and pentraxin-related gene (*PTX3*).

We also mapped the SAM-selected differentially expressed genes onto a cell diagram to visualize the common regulators of the modified transcripts using Pathway Studio (Figure 2). Color intensity shows the level of abundance of each transcript (red, increased; green, decreased) in lymphocytes from the high-arsenic exposure compared with the low-arsenic-exposure group. The lines represent relationships among the top-ranked biologic molecules that have been established by previous work published in the literature. Importantly, this diagram helps predict differential expression of the genes that may have profound biologic consequences (i.e., by modulating protein stability or activation level) that are not observable in a gene expression microarray.

Using an alternative analysis strategy to more broadly characterize arsenic-associated expression modification, we used EVA software to graphically characterize functions of the top ranked 12,484 genes selected by ReliefF algorithm. Thus, we are including many genes with more subtle or less consistent regulation than in the SAM-selected set. This technique detected an overrepresentation of genes (Fisher exact *p* < 0.05) involved in the KEGG biopathway and Gene Ontology processes, including 7 in natural killer cell–mediated cytotoxicity and cellular defense response, 8 in antigen processing and presentation and immune response, 12 cellular adhesion molecules (CAMs) and cell adhesion, and 6 in the Wnt signaling pathway and regulation of transcription. Other high-ranking but nonsignificant categories included regulation of actin cytoskeleton, neuroactive lig-and–receptor interaction, Jak-STAT signaling, axon guidance, ABC transporters, peroxisome proliferator–activated receptor (PPAR) signaling pathway, and type 1 diabetes mellitus.

In addition, we performed an analysis restricted to the subset of 10 individuals with data on high versus low urinary arsenic levels. In this way, we avoid any potential misclassification of individuals that could be associated with relying on home drinking-water arsenic concentration or toenail levels, which are longer term biomarkers of exposure than urine. We compared expression levels for individuals with a urinary biomarker of very recent high arsenic exposure versus those with low urinary arsenic exposure using SAM. We identified a total of 38 genes that were significantly different in those with high versus low urinary arsenic levels [Supplemental Material, Table 2 (http://www.ehponline.org/members/2008/10861/suppl.pdf)]. We used DAVID functional annotation clustering to assess the statistically significant overrepresentation of these urinary arsenic–modified genes by biological category: natural killer cell–mediated cytotoxicity (*p* < 0.001), immune response (*p* < 0.001), antigen processing and presentation (*p* < 0.001), and apoptosis (*p* = 0.009).

**Figure 3:**
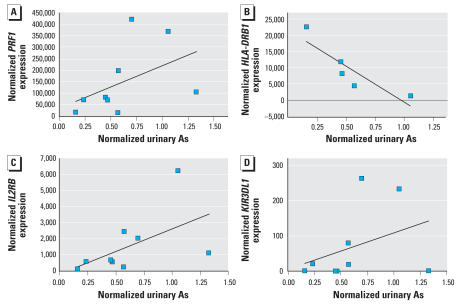
RT-PCR analysis of gene expression in relation to urinary arsenic (As). The graphs depict the normalized RT-PCR gene expression versus creatinine-normalized urinary arsenic exposure levels. (*A*) *PRF1* (*r*^2^ = 0.2). (*B*) IL2RB (*r*^2^ = 0.3). (*C*) *HLA-DRB1* (*r*^2^ = 0.8). (*D*) *KIR3DL1* (*r*^2^ = 0.1).

Real-time PCR validation using independent primer sets selected based on the micro-array results are shown in Figure 3. Relative expression levels of each gene in a lymphocyte sample are plotted against the creatinine-normalized urinary arsenic level for that subject. Despite substantial variability, there was a trend toward increased transcript abundance for *PRF1*, *IL2RB*, and KIR3DL1 with increasing urinary arsenic concentration. HLA-DRB1 expression significantly decreased with higher urinary arsenic levels (*r*^2^ = 0.8, *p* < 0.05).

## Discussion

Chronic arsenic exposure at levels found in U.S. drinking water has been associated with cancer, cardiovascular disease, and diabetes (Engel et al. 1994; Karagas et al. 2001, 2004; Meliker et al. 2007; Steinmaus et al. 2003). Our microarray-based genomewide analyses detected patterns of decreased/increased transcript abundance in peripheral blood lymphocytes from the high- versus low-exposure groups representing North American drinking-water arsenic levels. The biologic functions of the transcripts with statistically significant differences included an overrepresentation of genes involved in defense response, immune function, and apoptosis. Although these observed changes in lymphocytes may be interpreted as surrogate markers for changes that occur in other cell types, they also directly reflect biologically important changes in the blood of arsenic-exposed individuals that may be directly involved in disease. For example, inhibition of immune function may play a role in promoting immune escape and tolerance of tumors (LeMaoult et al. 2005).

Despite differences in overall arsenic levels and country of origin among the previously reported studies of arsenic-exposed human lymphocytes, there are similarities in the differentially abundant transcripts between the high-and low-arsenic-exposed populations. Our data suggest decreases in defense response genes, including heat-shock proteins. Similarly, heat-shock protein *HSPA1B*/*HSP70* expression was modified in lymphocytes from arsenic-exposed individuals from Bangladesh, as well as in several animal and cell culture studies, probably in response to the generation of reactive oxygen species (Andrew et al. 2003, 2007; Han et al. 2005; Liu et al. 2001). The Bangladesh and Taiwan studies and our U.S. study all observed a decrease in inflammatory response pathway members, including interleukins such as *IL2R* (Argos et al. 2006) and *IL1* β (Wu M.M. et al. 2003). Argos et al. (2006) reported differential expression of *USP13* and the ubiquitin-conjugating enzyme *UBE2E1*, and we also saw differences in several ubiquitin cycle transcripts.

Our human data also strongly support previous results from animal and *in vitro* studies indicating that chronic arsenic exposure modulates immune function (IARC 2004). Mice treated with arsenic had increased bacterial load accompanied by decreased adhesion, chemotactic migration, and phagocytic ability of splenic macrophages (Lewis et al. 1998). Exposure to 2 and 10 μg/L arsenic suppressed innate immune response in a zebrafish model, increasing pathogen load and reducing respiratory burst activity (Nayak et al. 2007). *In vitro* arsenic exposure (< 75 μg/L) inhibited maturation of monocytes to macrophages by producing an abnormal nonadhesive macrophage and reorganization of the actin cytoskeleton. These arsenic-exposed macrophages had higher granulocyte-macrophage colony-stimulating factor–stimulated MHC class II molecule HLA-DR protein levels, and decreased phagocytic ability (Lemarie et al. 2006; Sakurai et al. 2005, 2006).

We observed decreased abundance of CD69 in lymphocytes from the high-arsenic group, an effect that was observed previously at the protein level in CD4^+^ and CD8^+^ lymphocytes treated with arsenic *in vitro* (Conde et al. 2007; Tenorio and Saavedra 2005). Our highly exposed lymphocytes also had increased levels of *IL2RB*, which is necessary for activation of STAT5-dependent T-regulatory cell differentiation (Burchill et al. 2007). Similarly, Soto-Pena et al. (2006) reported reduced peripheral blood mononuclear cell mitogenic response and a modified CD4/CD8 lymphocyte cell ratio in children with high urinary arsenic levels. Moreover, mice exposed to 50 mg/L arsenite in their drinking water had inhibited T-lymphocyte proliferation in response to mitogens (Patterson et al. 2004). *In vitro* studies indicated that arsenic delayed proliferation of T-lymphocytes and modified DNA synthesis in a biphasic dose-dependent manner (Galicia et al. 2003; Meng and Meng 1994).

Natural killer cells act as a first line of defense in the blood and are also recruited to mucosal tissues with inflammation or infection, including the lung (Sentman et al. 2007). In our study, lymphocytes from the high-arsenic-exposure group had increased levels of a number of inhibitory killer cell immunoglobulin-like receptors, as well as the killer cell lectin-like receptors *GZMB*, *PRF1*, and *NFATC3* [Table 1; Supplemental Material, Figure 1 (http://www.ehponline.org/members/2008/10861/suppl.pdf)]. KIR genes are expressed by natural killer cells as well as memory α BT cell, serving both innate and adaptive immune response (Parham 2004). GZMB and PRF1 help the induction of apoptosis via the cytotoxic T-lymphocytes or natural killer cells in cell-mediated immune response (Adrain et al. 2006; Veugelers et al. 2006). GZMB specifically destabilizes the cell cytoskeleton during cytotoxic T-lymphocyte and natural killer cell–mediated cell killing (Adrain et al. 2006). NFATC3 is a transcription factor involved in T-cell development that is activated by hypoxia (Cante-Barrett et al. 2007).

In addition to the pervasive effects on immune response pathways, the lymphocytes from the high-arsenic-exposure group showed differences in transcripts involved in diabetes and nervous system development. Arsenic exposure has been associated with increased diabetes mellitus–related mortality in several populations, including the United States (Chen et al. 2007; Meliker et al. 2007). Similarly, our study shows differences in transcripts involved in diabetes by arsenic exposure status. The high-arsenic-exposure group also had modified levels of several transcripts involved in the nervous system and other aspects of development, supporting associations between arsenic exposure and fetal and early childhood effects (Hopenhayn et al. 2006; Rahman et al. 2007; Wasserman et al. 2004). One of our novel findings is that lymphocytes from arsenic-exposed individuals had higher levels of cytochrome P450 2E1 (CYP2E1), which metabolizes and bioactivates a number of chemicals by forming epoxides, including benzene, vinyl chloride, 1,1-dichloroethylene, trichloroethylene, 1,3-butadiene, acrylonitrile, and acrylamide, and metabolizes acetaminophen (Ghanayem 2007).

To elucidate common regulators of the differentially abundant transcripts in the high-versus low-arsenic groups, we mapped our results on a literature-based pathway diagram (Figure 2). Notably, TP53 regulates intercellular adhesion molecule 1 (ICAM-1), which controls immune cell migration and adhesion, and interferon-related developmental regulator 1 (IFRD1/PC4), which regulates p53 transcriptional activity and is required for myoblast differentiation, and also inhibits MyoD and MADS box transcription enhancer factor 2, polypeptide C (MEF2C) (Batta and Kundu 2007; Gorgoulis et al. 2005; Micheli et al. 2005; Nery et al. 2006). PPARA-α activates the nuclear hormone receptor nuclear receptor subfamily 1, group D, member 2 (*NR1D2*), blocking signaling from the orphan receptor-α (ROR-α) that regulates high-density lipoprotein cholesterol, lipid homeostasis, and inflammation (Ramakrishnan et al. 2005). E1A binding protein p300 is a common regulator of the transcription factor PPARA-α and the transcription factors *MEF2C* and signal transducer and activator of transcription 2 (*STAT2*) (Bhattacharya et al. 1996; Ma et al. 2005). The Rho-family small G-protein cell division cycle 42 homolog *(Saccharomyces cerevisiae)* (*CDC42*), controls progression through the cell cycle and also helps regulate degradation of the central carcinogenesis mediator, the epidermal growth factor receptor (EGFR) (Wu WJ et al. 2003). These human data suggest that future experimental studies of the regulation of these pathways by arsenic may provide insights into mechanisms of arsenic toxicity.

One possible limitation of this study is that the proportion of smokers varies slightly by arsenic exposure. This difference in smoking status is not driving the expression of genes selected for this analysis, because the gene expression changes are observed regardless of smoking status. Furthermore, there is abundant evidence that the most important health effects of arsenic may occur in combination with smoking (Chen et al. 2004; Rossman et al. 2002).

Our study is the first to investigate genomewide gene transcript abundance differences that occur in individuals exposed to drinking-water arsenic contamination in the United States. The modulated pathways, including defense response, immune function, cell growth, apoptosis, regulation of cell cycle, T-cell receptor signaling pathway, and diabetes, were consistent with previous reports from arsenic-exposed populations. Many of the genes are involved in carcinogenesis, diabetes, and immunosuppression, which are previously documented health effects of chronic arsenic exposure. Future studies are needed to elucidate the mechanisms through which chronic exposure to arsenic modulates expression of the identified genes, to clarify the direct roles of changes in the lymphocytes themselves in contrast with surrogate changes that also occur in other tissues, and to understand the health consequences of these changes. The arsenic-modulated pathway members identified in the pathogenesis of these diseases are potential targets for mechanistic studies and prophylactic or chemopreventive treatment and are candidates for biomonitoring of individuals with a history of arsenic exposure.

## Figures and Tables

**Table 1:** Immune response genes significantly associated with arsenic exposure level.

Gene symbol (LocusLink)/ Probe ID^a^	Gene name	Fold change	Gene ontology terms^a^	KEGG pathway^b^
*HLA-DQA1* (3117)	major histocompatibility complex, class II, DQ alpha 1			
203290_at		3.37	APEA, IR	APAP
236203_at		0.38		
213831_at		0.31		
*KIR3DL2* (3812)	killer cell immunoglobulin-like receptor, three domains, long cytoplasmic tail, 2			
207314_x_at		2.25	CDR	APAP, NKMC
211688_x_at		1.75		
216907_x_at		1.65		
207313_x_at		1.45		
*SH2D1B* (117157)	SH2 domain containing 1B			
1553177_at		2.03	NKMC	
*KIR3DL1* (3811)	killer cell immunoglobulin-like receptor, three domains, long cytoplasmic tail, 1			
211389_x_at		1.88	IR, NKCA, NRNK, MHCI, HBSI	APAP, NKMC
*KIR2DL3* (3804)	killer cell immunoglobulin-like receptor, two domains, long cytoplasmic tail, 3			
208179_x_at		1.82	AB, IR	APAP, NKMC
*KIR2DL1* (3802)	killer cell immunoglobulin-like receptor, two domains, long cytoplasmic tail, 1			
210890_x_at		1.78	NRNK, HCSI, IR	APAP, NKMC
*KIR2DL2* (3803)	killer cell immunoglobulin-like receptor, two domains, long cytoplasmic tail, 2			
211397_x_at		1.74	IR	APAP, NKMC
*PRF1* (5551)	perforin 1 (pore forming protein)			
1553681_a_at		1.74	CDR, PATH, VICA	NKMC
214617_at		1.53		
*KIR3DL3* (115653)	killer cell immunoglobulin-like receptor, three domains, long cytoplasmic tail, 3			
216676_x_at		1.68	APAP	
*KIR2DS5* (3810)	killer cell immunoglobulin-like receptor, two domains, short cytoplasmic tail, 5			
208203_x_at		1.57	IR, HCSI	APAP
*RAC2* (5880)	rho family, small GTP binding protein Rac2			
207419_s_at		1.55	NKMC, BCRS, FCRI, LTM	
*KIR2DL5A* (57292)	killer cell immunoglobulin-like receptor, two domains, long cytoplasmic tail, 5A			
211410_x_at		1.49	APAP, NKMC	
*CD247* (919)	CD247 molecule			
210031_at		1.48	TCRC	NKMC, TCRS
*KLRF1* (51348)	killer cell lectin-like receptor subfamily F, member 1			
220646_s_at		1.43	AHR, MHCI	
*GBP5* (115362)	guanylate binding protein 5			
238581_at		1.41	IR	
*KLRK1* (22914)	killer cell lectin-like receptor subfamily K, member 1			
205821_at		1.38	NKMC	
*KIR2DL4* (3805)	killer cell immunoglobulin-like receptor, two domains, long cytoplasmic tail, 4			
211242_x_at		1.36	CDR	APAP, NKMC
211245_x_at		1.25		
208426_x_at		1.24		
*KIR2DS1* (3806)	killer cell immunoglobulin-like receptor, two domains, short cytoplasmic tail, 1			
208198_x_at		1.34	IR	APAP, NKMC
*NFATC3* (4775)	nuclear factor of activated T-cells, cytoplasmic, calcineurin-dependent 3			
207416_s_at		1.33	INFR	NKMC, TCRS, BCRS
*DKFZP564J0863* (25923)				
224893_at		1.2	IR	
*TAL1* (6886)	T-cell acute lymphocytic leukemia 1			
216925_s_at		0.83		
*SOS1* (6654)	son of sevenless homolog 1 (*Drosophila*)			
212780_at		0.83	NKMC, TCRS, FCRI	
*MALT1* (10892)	mucosa associated lymphoid tissue lymphoma translocation gene 1			
210017_at		0.82	DR	TCRS
*HLA-DQB1* (3119)	major histocompatibility complex, class II, DQ beta 1			
212998_x_at		0.54	IR	APAP
211654_x_at		0.51	APAE	
212999_x_at		0.35	APEA	
209480_at		0.28	MHCII	
*CDC42* (998)	cell division cycle 42 (GTP binding protein, 25 kDa)			
214230_at		0.53	MD	TCRS, LTM, ECSH
*HLA-DPA1* (3113)	major histocompatibility complex, class II, DP alpha 1			
213537_at		0.49	IR, APEA	APAP
*PTX3* (5806)	pentraxin-related gene, rapidly induced by IL-1 beta			
206157_at		0.35	INFR	

Gene ontology abbreviations: AB, antigen binding; AHR, antimicrobial humoral response (sensu Vertebrata); APAE, antigen presentation, exogenous antigen; APEA, antigen processing, exogenous antigen via MHC class II; CDR, cellular defense response; DR, defense response; HBSI, human leukocyte antigen-B (HLA-B) specific inhibitory MHC class I receptor activity; HCSI, HLA-C specific inhibitory MHC class I receptor activity; INFR, inflammatory response; IR, immune response; MD, macrophage differentiation; MHCI, MHC class I receptor activity; MHCII, MHC class II receptor activity; NKCA, natural killer cell activation; NRNK, negative regulation of natural killer cell activity; PATH, pathogenesis; TCRC, T-cell receptor complex; VICA, virus-infected cell apoptosis.

b KEGG pathway abbreviations: APAP, antigen processing and presentation; BCRS, B-cell receptor signaling pathway; ECSH, epithelial cell signaling in *Helicobacter pylori* infection; FCRI, Fc epsilon RI signaling pathway; LTM, leukocyte transendothelial migration; NKMC, natural killer cell–mediated cytotoxicity; TCRS, T-cell receptor signaling pathway.

a Probe IDs were obtained from the Affymetrix NetAffx database (http://www.affymetrix.com/analysis/index.affx).
